# Assessemnt of nasal bone in first trimester screening for chromosomal abnormalities in Khuzestan 

**Published:** 2014-05

**Authors:** Sara Masihi, Mojgan Barati, Razieh Mohamadjafari, Marzieh Hashemi

**Affiliations:** 1 *Depatment of Obstetric and Gynecology, Fertility Infertility and Perinatology Research Center, Ahvaz Jundishapour University of Medical Sciences, Ahvaz, Iran.*; 2 *Department of obstetrics and Gynecology, Ahvaz Jundishapour University of Medical Sciences, Ahvaz, Iran.*

**Keywords:** *Prenatal diagnosis*, *Nuchal translucency measurement*, *Maternal serum screening test*, *Nasal bone*

## Abstract

**Background:** Fetal nasal bone assessment is a non-invasive procedure that helps provide even greater assurance to patients undergoing their first trimester risk assessment for aneuploidies. Absence or presence of this factor is different in some races.

**Objective:** The study was aimed to evaluate nasal bone in the first trimester of pregnancy in the indigenous population of Khuzestan Province, and to monitor its value in the diagnosis of chromosomal abnormalities.

**Materials and Methods: **This study was conducted on 2314 pregnant women between 17-43 years old who referred for first trimester screening for chromosomal abnormalities. Gestational age was between 11-13w + 6 days. Nuchal translucency (NT), fetal heart rate (FHR), crown rump length (CRL), and maternal age and maternal blood serum factors (Free HCG) and pregnancy-associated plasma protein-A (PAPP-A) and nasal bone were assessed. Finally the risk of trisomies was calculated. The statistical tests are based on the relationship between chromosomal abnormality and the presence or absence of the nasal bone.

**Results: **In 114 cases we could not examine the nasal bone. Also, in 20 cases missed abortion happened without knowing the karyotype. 2173 cases were delivered normal baby, and in seven cases chromosomal abnormalities were diagnosed. Nasal bone was absent in all three cases with trisomy 21 and six of 2173 cases with normal phenotype (0.3%). With use of the Fisher exact test (p=0.0001), a significant correlation was found between the absence of the nasal bone and the risk of chromosomal abnormality.

**Conclusion:** Inclusion of the nasal bone in first-trimester combined screening for aneuploidies achieves greater detection rate especially in Down syndrome.

## Introduction

Down's syndrome is one of the most common clinical syndromes with prevalence of one in every 800-1000 live births (1). This syndrome is one of the few syndromes that fetus survives after birth, but it is accompanied with great difficulties. This issue imposes a lot of emotional and economic costs to society. There are several screening tests for the detection of chromosomal abnormalities in the first trimester of pregnancy, including measurement of nuchal tranclucency (NT) in 11-14 weeks. Also, serum markers the free βHCG and PAPP-A in combination with NT are other screening markers of chromosomal abnormalities. Furthermore, the absence of the nasal bone or its hypoplasia is one of the sonographical markers for helping the diagnosis of Down's syndrome. In 2001, it was found that the nasal bone is absent in 60-70% of the fetuses with Down's syndrome and 2% of normal fetuses in 11-14 weeks ultrasound. Anthropometric studies have shown that nasal root depth in 50% of cases of Down's syndrome is abnormally low (3). Nicolaides *et al* collected the results obtained in studies of 15,822 fetuses in which 97.4% of cases have been well evaluated, and did not observe nasal bone in 1.4% of normal fetuses and 69% of fetuses with trisomy 21 (2). 

An important finding is the absence rate of the nasal bone decreases with increased crump-lump lenght and increases with increasing NT. Also, the rate in African Caribbean is higher than whites (3). In a study conducted by Nicolaides *et al* in Great Britain, guidelines for fetal screening at 11-13 weeks of gestation were studied (3). This study showed that the combination of serum factors (Free βHCG pregnancy-associated protein A) and NT to 90% of fetuses with trisomy 21 are identified. If in addition to the NT and serum markers, other sonographical markers such as the presence of the nasal bone, doppler flow in the Ductus Venosous or in the hepatic artery and or the blood through the tricuspid valve to be used, the detection rate for Down's syndrome increases to 95%. 

In a study conducted in 2010 by Schluter *et al* in Australian universities, there was a significant difference in the rate and length of nasal bone in the Asian and Caucasian descent population (4). In a study conducted in 2010 in Turkey by Ozer *et al*, it was found that the presence of nasal bone in the first trimester of pregnancy has a direct relationship with the CRL as well as the patient's GA; there was no significant difference between the indigenous population of Turkey and Caucasian descent population (5). In 2009, a study was conducted by Kagan *et al *in German universities in which the presence or absence of the nasal bone screening in the diagnosis of Turner syndrome and trisomy 18, 21, and 13 was investigated and the following results were obtained: Nasal bone were not observed in 2.6% of euploid embryos but in the trisomy 21, trisomy 18, and trisomy 13 were absent 59.8%, 52.8%, and 45%, and also in all of the fetuses with Turner’s syndrome (6). 

**Figure 1:**
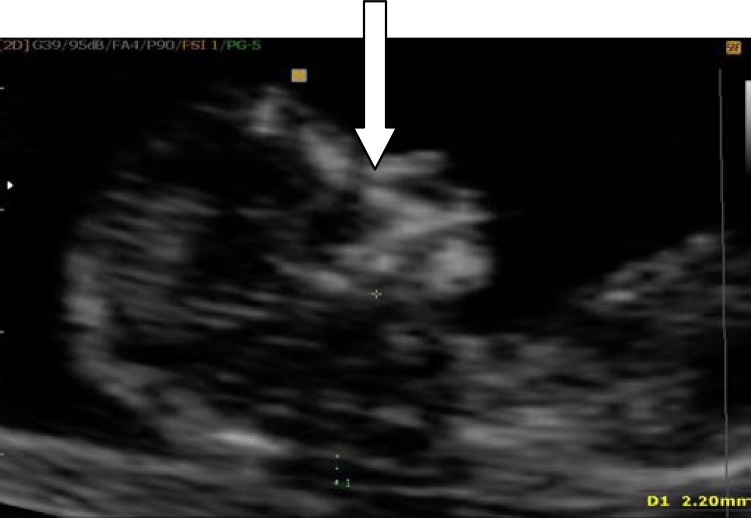
Nasal bone in sagittal view

Factors for maternal age and NT, and fetal heart rate (FHR) and serum free βHCG and PAPP-A were used in this screening; based on these factors and the cut of risk 1 in 100, false positive rate was 3%, if the nasal bones factor added to screening, the false positive rate was declined to 2.5%. Another study in 2003 by Bunduki *et al* was conducted in São Paulo university in Brazil where 1,631 women over 35 years at 16-24 weeks of pregnancy of gestation were examined and the length of the nasal bones less than fifth percentile of the population was identified as the cut of risk. 1631 singleton pregnancy cases and mothers over 35 years were investigated in this study and a linear relationship was discovered between the presence or absence of the nasal bone and Down's syndrome (Figure 1) (7). This study has been planned to evaluate the importance of fetal nasal bone in the diagnosis of choromosomal abnormalities in khuzestan province.

## Materials and methods

This was a descriptive-analytical epidemiological study in which all pregnant women referred to Imam Khomeini and Razi hospitals in Ahvaz for prenatal care underwent the first trimester screening between May 2012 to May 2013. Inclusion criteria in the study design were pregnant women at all age in the first trimester of pregnancy at the gestational age of 11-13 and 6day weeks. Exclusion criteria were abortion, and a lack of accessibility to the karyotype of aborted fetus. Pregnant women underwent first trimester ultrasound, NT, CRL, and FHR; and the presence or absence of the nasal bone was investigated. The patients also simultaneously were investigated for the serum markers free βHCG and pregnancy associated plasma protein A (PAPP-A). 

These values entered in FMF software and risk of trisomies calculated, and based on adjusted risk (risk of chromosomal abnormalities were classified into three groups: 1: low Risk 2: high risk and 3: moderate risk). Individuals belonging to the high-risk group, i.e. people with cut of risk greater than or equal to one percent will be offered invasive testing. Low-risk patients, i.e. those with a modified risk less than one per thousand underwent the routine pregnancy and continued screening in the second trimester. Moderate risk group who had risk between 1% to 1/1000 had benefit after assessment of nasal bone. 

Absence or presence of nasal bone converted the intermediate group into either low risk or high-risk groups. Finally, the invasive testing offered to individuals in high-risk groups was followed up, and the karyotype test of the fetus was considered for them. The people in the low-risk group were also followed during pregnancy, and the pregnancy outcome was evaluated in terms of the presence or absence of chromosomal abnormalities in the fetus based on the phenotype. At the end, the relationship between the absence of nasal bone and the risk of chromosome abnormalities were determined by Fisher's exact test. Also, the amount of the relative frequency of the being positive factor in the population under study were determined. It is noticeable that consents were given by all cases after consoling. The study was accepted by ethical committee of university.

## Results

**Figure 2:**
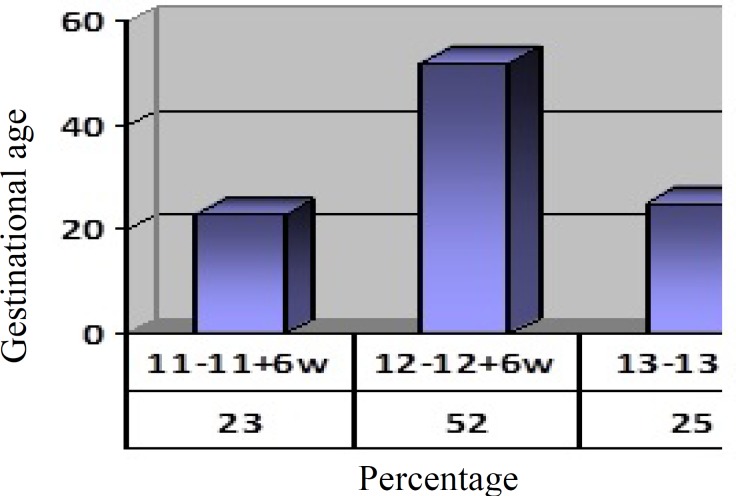
Percentage of subjects according to gestational age

Of the 2314 women under study of nasal bone factors, 114 patients were no evaluable (a profile of fetal face was not of good quality for examining nasal bone). A total of 20 cases were aborted, which fetal karyotype was not evaluable in these cases. A total of 2180 pregnant women in whom fetal nasal bone was examined, the number of 2173 cases was of fetuses with normal phenotype which included %99/7 of cases. And 7 cases had a chromosomal abnormality which included 0.3% of cases. GA of the subjects was 11-13^+6^ weeks; Figure 2 shows the separate percentage. The CRL was between 45 and 84 mm (middle the 63.2 mm). 

In 3 cases of trisomy 21, CRL was 60, 71 and 83 mm, respectively. In 2 cases of trisomy 18, CRL was 61.6 and 63 mm, respectively. In case with trisomy 13, the CRL was 60 mm; and for Turner syndrome, CRL was 82 mm. NT measurement was changing from 0.8-9.8 mm, and it’s medium was 1.5 mm. 67.6% of patients were in the medium range, and 3.7% of the population had the NT higher than 95%. There was significant relationship between absence of nasal bone with abnormal NT (p=0.007). In other words, in embryos which had nasal bone negative factor, the proportion of abnormal NT was 27.3% vs. 3.6% in the embryos in which nasal bone was observed (Tables II and V).

Serum markers of women under study: measurement of serum markers was done just on 956 cases due to a lack of devices to evaluate these tests in earlier cases in the centers under our study. Of these, in 4.8% of women free βHCG level was above 95% of percentile, and in 7.2% of the cases PAPP-A was below 5% of percentile. Fetal anomaly did not discover in none of the women in these two groups. In women who had fetal anomaly unfortunately only one case checked the serum markers, trisomy18 was detected in a woman 35 years old with CRL61.6 and NT 2.6 mm and the adjusted risk 1: 55 that serum markers were in the normal scope (Tables III and V). 

The adjusted risk of under study: in this study, out of 2173 cases with embryos having normal phenotype, 83 cases (3.8%) have been rated at a high risk of 1:100. 11.4% have been rated at a risk 1:100 up to 1:1000; 85% of have been with risk less than 1000. Among three cases of trisomy 21 that had the risk 1:7 and 1:131 and 1:340, respectively, 33.3% of have been with a risk greater than 1:100, and 66.7% of people were with a risk of 1:100 to 1:1,000. A person, who was risk of 1:7, underwent chorionic villus sampling (CVS); Down's syndrome was detected; and pregnancy terminated. Two other patients who had a risk of 1:131 and 1 in 340, due to the absence of nasal bone on ultrasonography, were placed in high-risk groups; they underwent the CVS, Down syndrome was diagnosed and also both pregnancies were terminated.

Among the two cases of trisomy 18, the risk was 1: 5 and 1: 55; they were placed in high risk groups; the nasal bones were not found in one of these cases. A person who had a risk 1: 5, was with nasal bone (NB+); and the person who rated the risk of 1: 55, nasal bone factor in her fetus was absent. In a woman who had a risk 1: 55, according to the result of the CVS and detection of trisomy 18, pregnancy termination was performed in the first trimester. But in a person with a risk 1:5 due to the lack of consent of the patient in order to perform the CVS at the first trimester, she underwent amniocentesis because of multiple anomalies in second trimester, and termination of the pregnancy was performed with regard to the result of the karyotype sampling.

For trisomy 13, which a case in this study was identified, modified risk for patient was 1: 3026, and it was in the low risk group; the nasal bones factor of the patient was positive; the patient was not diagnosed in the first trimester of pregnancy; and in the second trimester, she underwent amniocentesis due to severe hydrops; and pregnancy termination was performed according to the result of karyotype, which was trisomy 13. One case was TS; the adjusted risk was 1: 2, and she was in high risk groups; as the nasal bones factor in her fetus was negative, she underwent CVS, and was diagnosed with TS and the termination of pregnancy was performed. Assessment of nasal bone in our study: out of 2173 cases with a normal phenotype, in six cases nasal bone was absent (0.3%), and in (99.7%) NB was present. All three cases of trisomy 21 did not have nasal bone in sonographic images; among two cases of trisomy 18, in one case NB was absent, and in another case it was present. In a case of trisomy 13 nasal bones was visible. In a case of TS of nasal bone could not be seen. Using the Fisher exact test for correlation analysis, a significant relationship was observed between the lack of nasal bone and the chromosomal abnormalities (p=0.0001). In other words, the ratio of chromosomal abnormalities in fetuses without nasal bone was 3.9% vs. 0.1% if there was present nasal bone (This relationship was not evaluated for each chromosome abnormality alone due to the low number of cases) (Table IV).

**Table II:** Relationship between nuchal translucency and nasal bone in subjects

**Nasal bone factor**	**NT**		**Total**
	**Normal (n)**	**Abnormal**	
Present	2091	78	2169
Absent	8	3	11
Total	2099	81	2180

**Table III:** Serum markers of subjects

**Serum markers**	**5** ^th^ ** percentile**	**Median**	**95** ^th^ ** percentile**	
free βHCG (MOM)	0.265	0.833	2.584	4.8% (above the 95^th^ percentile)
PAPP-A (MOM)	0.385	1.02	2.556	72% (below the 5^th^ percentile)

PAPP-A: pregnancy associated plasma protein A

**Table IV:** Relationship between nasal bone and chromosomal abnormalities

**Nasal bone factor**	**Chromosomal abnormalities**		**Total**
	**Has**	**Has not**	
Present	2	2167	2169
Absent	5	6	11
Total	7	2173	2180

**Table V:** Profile of women having a child with chromosomal abnormalities

**Variable **	**Person 1**	**Person 2**	**Person 3**	**Person 4**	**Person 5**	**Person 6**	**Person 7**
Age	39	28	26	35	24	30	29
CRL (mm)	71	60	83	61.6	63	60	82
NT (mm)	1.9	2.7	5.2	2.6	8	1.6	9.8
hCG (MoM)	-	-	-	1.343	-	-	-
PAPP-A (MoM)	-	-	-	0.626	-	-	-
Estimated. risk	1:340	1:131	1:7	1:55	1:5	1:3026	1:2
Karyotype	Trisomy 21	Trisomy 21	Trisomy 21	Trisomy 18	Trisomy 18	Trisomy 13	Turner syn

Papp-A: pregnancy associated plasma protein A

## Discussion

The first trimester screening in pregnancy is very effective in on diagnosing the chromosomal abnormalities in our region, and assessment of nasal bone is one of effective secondary factors in this area. In our study, the lack of observing the nasal bone had a positive relationship with chromosomal abnormalities in the fetus; this result is similar to the results of Yeo *et al* study in Singapore; they concluded the use of nasal bone factor upgraded chromosomal abnormality diagnosis from 81.8-90.9% (8). Similarly Nicolaides in 2011 concluded that as well as serum markers and NT, if nasal bone factor be used in diagnosing chromosomal abnormalities, diagnostic accuracy increases from 90-95% (3). In another study conducted by Ghaffari and colleagues in Iran on over 13,437 women, ultrasound secondary factors including the nasal bone, tricuspid regurgitation (TR), and ductus venosus flow (DV) accompanied by NT and serum markers have increased accuracy of diagnosis of chromosomal abnormalities, and have decreased positive rate (FPR) from 4.8-3.4% (9). 

In the study, nasal bone factor in 99.7% of fetuses with normal phenotype was present, and in 0.3% of fetuses was absent which was the equal to the world statistics. In world statistics, rate of positive nasal bone factor in the white race in a fetus with a normal karyotype is from 98-9.5% has been reported. And nasal bones cannot be seen in 0.5-2% of ffetuses with a normal karyotype. While in various statistics based on Nicole ides study, nasal bone was absent in 60-70% of fetuses with trisomy 21 (10). In this study, nasal bone factor was not seen in any three cases of trisomy 21, but due to the low number of cases of trisomy 21, it cannot be compared to global statistics. Among two trisomy’s 18, one was with present nasal bone and the other was with absent nasal bone; in the other studies conducted by Kagen and Nicoleides nasal bone factors in 50% of cases of trisomy 18 were absent; although this relationship is also due to the low number of cases which maybe arguable. In this study, in one case with trisomy 18 nasal bone was absent; in global statistics, in 70% of cases, this factor is present, and in 30% of cases is absent (6, 11). 

In this study, the nasal bone was also negative in case with Turner syndrome. In the cases with Down's syndrome, with respect to the persons with adjusted risk, which were 1:7, 1:131 and 1:340, respectively, assessment of nasal bone has helped us to detect Down's syndrome. Considering the two of cases with intermediate risk, after assessment of nasal bone, they were placed in the high risk group, and underwent invasive test and Down syndrome was detected. The interesting thing about trisomy 13 in this study was that a woman aged 30 years had a fetus with CRL 60 and NT 1.6 mm and a adjusted risk of 1-3026 that fetal trisomy was not diagnosed by any of the factors mentioned in the first trimester of pregnancy. 

This shows weakness of the software FMF in diagnosing syndrome 13, and it will lead us toward further effort in this area. About the indigenous people of Khuzestan Province in the software system of FMF, it seems that the people of the area are classified under the white race because the factor of nasal bones in 0.2-2% of people with a normal karyotype is not found in the global statistics (10). In this study, it became clear that nasal bone is present in 0.3% of patients with normal fetuses.

Finally, another remarkable thing was that among the people (seven cases) in which fetal anomalies were detected, only one patient was 39 years and all pregnant women with fetuses with chromosomal abnormalities were under 35 years of age. And this event leads us towards screening all pregnant women for fetal anomalies increasingly and necessitates more accurate and more comprehensive tests based on the race and the environment in which one grows. Finally, it is noteworthy that more accurate results and a more comprehensive study with a larger sample size are necessary.

## Conclusion

Given the high costs of having children with fetal abnormalities and mental and financial burden on the family, so, the early diagnosis of chromosomal abnormalities, including Down's syndrome is a step toward reducing these costs; based on the characteristics of environment and the local people, each country is to find appropriate solutions and every country and region should find appropriate strategies in this regard. Assessment of nasal bone associated with other ultrasound factors in the area under our study is an effective step to reduce false negative results and accurate diagnosis of this syndrome. 
